# Antidepressant-Like Effect of Terpineol in an Inflammatory Model of Depression: Involvement of the Cannabinoid System and D2 Dopamine Receptor

**DOI:** 10.3390/biom10050792

**Published:** 2020-05-20

**Authors:** Graziela Vieira, Juliana Cavalli, Elaine C. D. Gonçalves, Saulo F. P. Braga, Rafaela S. Ferreira, Adair R. S. Santos, Maíra Cola, Nádia R. B. Raposo, Raffaele Capasso, Rafael C. Dutra

**Affiliations:** 1 Laboratory of Autoimmunity and Immunopharmacology (LAIF), Department of Health Sciences, Campus Araranguá, Universidade Federal de Santa Catarina, Araranguá 88906-072, Brazil; grazielavieira@live.com (G.V.); julicavalli28@gmail.com (J.C.); elainecdalazen@gmail.com (E.C.D.G.); mcola1@yahoo.com.br (M.C.); 2 Post-Graduate Program of Neuroscience, Center of Biological Science, Campus Florianópolis, Universidade Federal de Santa Catarina, Florianópolis 88040-900, Brazil; adair.santos@ufsc.br; 3 Laboratório de Modelagem Molecular e Planejamento de Fármacos, Departamento de Bioquímica e Imunologia, Universidade Federal de Minas Gerais, Belo Horizonte 31270-901, Brazil; saulobragaqf@gmail.com (S.F.P.B.); rafaelasalgadoferreira@gmail.com (R.S.F.); 4 Laboratory of Neurobiology of Pain and Inflammation, Department of Physiological Sciences, Center of Biological Sciences, Universidade Federal de Santa Catarina, Florianópolis 88040-900, Brazil; 5 Center for Research and Innovation in Health Sciences (NUPICS), Faculty of Pharmacy, Universidade Federal de Juiz de Fora, Juiz de For a 36036-330, Brazil; nadiacritt@gmail.com; 6 Department of Agricultural Sciences, University of Naples Federico II, 80055 Portici, Italy; 7 Endocannabinoid Research Group, 80078 Naples, Italy

**Keywords:** depression, terpenoids, phytocannabinoid, cannabinoid receptor, dopaminergic receptor

## Abstract

Depression has a multifactorial etiology that arises from environmental, psychological, genetic, and biological factors. Environmental stress and genetic factors acting through immunological and endocrine responses generate structural and functional changes in the brain, inducing neurogenesis and neurotransmission dysfunction. Terpineol, monoterpenoid alcohol, has shown immunomodulatory and neuroprotective effects, but there is no report about its antidepressant potential. Herein, we used a single lipopolysaccharide (LPS) injection to induce a depressive-like effect in the tail suspension test (TST) and the splash test (ST) for a preventive and therapeutic experimental schedule. Furthermore, we investigated the antidepressant-like mechanism of action of terpineol while using molecular and pharmacological approaches. Terpineol showed a coherent predicted binding mode mainly against CB1 and CB2 receptors and also against the D2 receptor during docking modeling analyses. The acute administration of terpineol produced the antidepressant-like effect, since it significantly reduced the immobility time in TST (100–200 mg/kg, p.o.) as compared to the control group. Moreover, terpineol showed an antidepressant-like effect in the preventive treatment that was blocked by a nonselective dopaminergic receptor antagonist (haloperidol), a selective dopamine D2 receptor antagonist (sulpiride), a selective CB1 cannabinoid receptor antagonist/inverse agonist (AM281), and a potent and selective CB2 cannabinoid receptor inverse agonist (AM630), but it was not blocked by a nonselective adenosine receptor antagonist (caffeine) or a β-adrenoceptor antagonist (propranolol). In summary, molecular docking suggests that CB1 and CB2 receptors are the most promising targets of terpineol action. Our data showed terpineol antidepressant-like modulation by CB1 and CB2 cannabinoid receptors and D2-dopaminergic receptors to further corroborate our molecular evidence.

## 1. Introduction

Currently, depression is considered to be a complex mood disorder, which affects nearly 16% of the population, and it is the third leading source of years lived with disability worldwide [1]. In turn, it is among the major public health problem, since it represents a substantial burden because of its high incidence and the costs related to this disorder [2,3]. Its clinical features, including persistent sadness and apathy, sleep disturbances, anhedonic effect, reduced social interest, anorexia, and sickness behavior [4,5]. Several hypotheses have emerged to explain the pathophysiology of depression such as monoaminergic (serotonin and noradrenaline) theory, which supports antidepressant prescription [6]. However, these drugs have failed to treat different aspects of depression, which suggests the involvement of other mechanisms, beyond monoaminergic theory, in the development of depression [7]. Thus, previous works have demonstrated that peripheral immune activation and neuroinflammation may contribute to the development of major depressive disorder [8]. Interestingly, the administration of SR141716A, which is a CB1 receptor antagonist, selectively increases monoaminergic neurotransmission, including dopamine, in the brain regions that are related to mood [9]. In animal studies, depressive-like behavior is observed after the administration of exogenous proinflammatory cytokines or a cytokine inducer, such as lipopolysaccharide (LPS) [10,11], which leads to sickness behavior. Based on the neuroinflammation hypothesis of depression, LPS treatment is extensively used as a tool to evaluate the underlying mechanisms of depression [10,11,12,13,14,15]. Around 24 h after LPS injection, rodents begin eating and moving similarly to control animals because their sickness behaviors have resolved. Pro-inflammatory cytokines will cause an elevation of indoleamine-2,3-dioxygenase (IDO) 24 h after LPS injection. During the same time that IDO is elevated, animals will be anhedonic and spend more time immobile in the forced swim test [16].

Monoterpenes are nonpolar compounds of low molecular weight [17] and terpineol is an important monoterpenoid alcohol present in the essential oils of several species of plants, such as *Eucalyptus* [18], *Artemisia princeps Pamp* [19], and *Cannabis sativa* [20]. This is a synthetic flavoring substance permitted by the Food and Drug Administration (FDA) for direct addition to food for human consumption, which has been mainly investigated by its anti-inflammatory and antifungal properties. In this context, terpineol mitigated the pro-inflammatory activity of LPS on human macrophages through modulation of nuclear factor (NF)-κB, p38 mitogen-activated protein kinase (p38-MAPK), or extracellular signal-regulated kinase (ERK) pathways [21]. Beyond anti-inflammatory properties, when combined with β-cyclodextrin (βCD), α-terpineol also demonstrated antinociceptive activity in an animal model of non-inflammatory chronic muscle pain model, which mimics fibromyalgia clinical features [22]. The authors established that the analgesic effect of this complex was reversed by systemic administration of naloxone or ondansetron. From this, it is possible to suggest that αTPN-βCD interacts with opioid (μ, κ, Δ) and 5-HT receptors, probably modulating the descending inhibitory pain system [22]. Furthermore, Parvardeh and colleagues showed that α-terpineol attenuated dependence and tolerance to the analgesic effect of morphine [23]. Altogether, it is achievable to hypothesize that terpineol could interact with different targets in the central nervous system (CNS), including receptors that are related to the pathogenesis of depression, such as serotonergic, dopaminergic, and adenosinergic receptors. Moreover, Ferber and colleagues suggested that terpenes, phytocannabinoid ligands, may be an important source for new candidates for the treatment of depression and anxiety disorders [24]. Herein, we performed a detailed investigation focused on the terpineol effects in the depressive-like behavior induced by LPS as well as its neuroprotective role. Additionally, aiming to gain insight into the possible mechanism of action of terpineol, molecular docking analysis—a key tool in structural molecular biology and computer-assisted drug design—were used to identify other possible targets to terpineol in CNS.

## 2. Materials and Methods 

### 2.1. Drugs and Reagents

The following drugs were used: LPS from *Escherichia coli* 0127:B8 (0.5 mg/kg, i.p.), terpineol (mixture of isomers, anhydrous: α-terpineol, ~73%; β-terpineol, ~6%; γ-terpineol, ~18%; 86480 code, CAS No. 8000-41-7) (100 mg/kg, p.o. and i.p.; and 200 mg/kg, p.o.), caffeine (3 mg/kg, i.p.), sulpiride (50 mg/kg, i.p.), haloperidol (0.2 mg/kg, i.p.), imipramine (20 mg/kg, i.p.), and propranolol (2 mg/kg i.p.) were obtained from Sigma Aldrich Company (St. Louis, MO, USA). AM281 (1 mg/kg, i.p.) and AM630 (1 mg/kg, i.p.) were purchased from Tocris Bioscience (Ellisville, MO, USA). All drugs were administered by the intraperitoneal (i.p.) route, with terpineol also being administered by oral gavage (p.o.) or i.p. routes. In a general way, the drugs were dissolved in saline, except for sulpiride that was diluted in saline with 5% DMSO [25], terpineol that was diluted in saline with 0.5% Tween 80 [26]. AM281 and AM630 were dissolved at 1 mg/mL in DMSO and 1% ethanol [27,28,29]. The final concentration of ethanol or DMSO never exceeded 5%, which did not affect *per se* in our protocols. 

### 2.2. ADMET Properties and Molecular Docking

Alpha-terpineol canonical simplified molecular input line entry specification (SMILES): CC1=CCC(CC1)C(C)(C)O was obtained from PubChem website in order to evaluate pharmacokinetic and toxicological parameters. The absorption, distribution, metabolism, excretion, and toxicity profile (ADMET) of the α-terpineol was predicted using the Admet SAR online server [30]. Molecular docking is a tool in structural molecular biology that is used to design computer-assisted drug. The goal of ligand-protein docking is to predict the predominant binding mode(s) of a ligand with a protein of known three-dimensional structure [31]. Docking studies were performed with the three-dimensional structures of cannabinoid receptors CB1 (PDB ID: 5XRA) and CB2 (PDB ID: 5ZTY) and dopaminergic receptor D2 (PDB ID: 6CM4) retrieved from the Protein Data Bank (PDB) [32,33,34]. The three-dimensional ligand structures that were used in this study were retrieved from the DrugBank database. Docking calculations were performed on GOLD 5.6.3 (Cambridge, CB2-1EZ, United Kingdom) while using Hermes 1.9.3 (Cambridge, CB2-1EW, United Kingdom) for visualization and the interactive docking setup [35]. Water molecules and ligands were removed from receptors before docking and a search box was established comprising residues within 8 Å of the co-crystalized ligands using the built-in protein preparation module. Default docking parameters were used with 200% search efficiency. Each molecule was subjected to a maximum of 100 docking runs and early termination was allowed if the top three solutions converged within a root-mean-square deviation (RMSD) range of 1.5 Å. The redocking studies were performed with crystallographic ligands from the aforementioned PDB structures, employing the scoring functions Goldscore and Chemscore. Docking of the second molecule of terpineol was performed while using the same protocol, except for treating the predicted pose on the first docking as part of the receptor. All of the results and interactions were analyzed on the Maestro interface (Schrödinger Small Molecule Discovery Suite, New York, NY, USA) and the figures prepared on Pymol.

### 2.3. Animals

The experiments were performed in male Swiss mice (20–30 g), 8–12 weeks old, obtained from the Universidade Federal de Santa Catarina. The animals were kept under a 12-h light/dark cycle (lights on at 7:00 a.m.) at temperature 22 ± 2 °C, with food and water *ad libitum* (maximum of 10 mice, group-housed). Animals were acclimatized to the laboratory settings for at least 1 h before testing and they were used only once throughout the experiments. The mice were randomly assigned before treatment or behavioral evaluation. All of the procedures used in the present study followed the “Principles of laboratory animal care” (NIH publication no. 85–23) and ARRIVE (Animal Research: Reporting of In Vivo Experiments) guidelines [36,37], as well as being approved by the Animal Ethics Committee of the Universidade Federal de Santa Catarina (CEUA-UFSC, protocol number 3914220319—approved in 05/07/2019). Moreover, the number of animals and the intensity of the noxious stimuli used were the minima necessary to demonstrate consistent effects. All of the experimental procedures were conducted according to the guidelines of CONCEA and CEUA/UFSC, based on the principles of the 3Rs (Replacement, Reduction, and Refinement). Mice were euthanized by cervical dislocation. Behavioral evaluations were performed between 8:00 a.m. and 5:00 p.m. All behavioral analysis was measured manually, and the observer was blinded to the experimental protocols.

### 2.4. Lipopolysaccharide (LPS) 

A single peripheral administration of LPS (0.5 mg/kg, i.p.) induced depressive-like behavior and we performed the behavioral analysis 24 h after LPS administration, according to a method previously described [38].

### 2.5. Experimental Design

**Figure 1:**
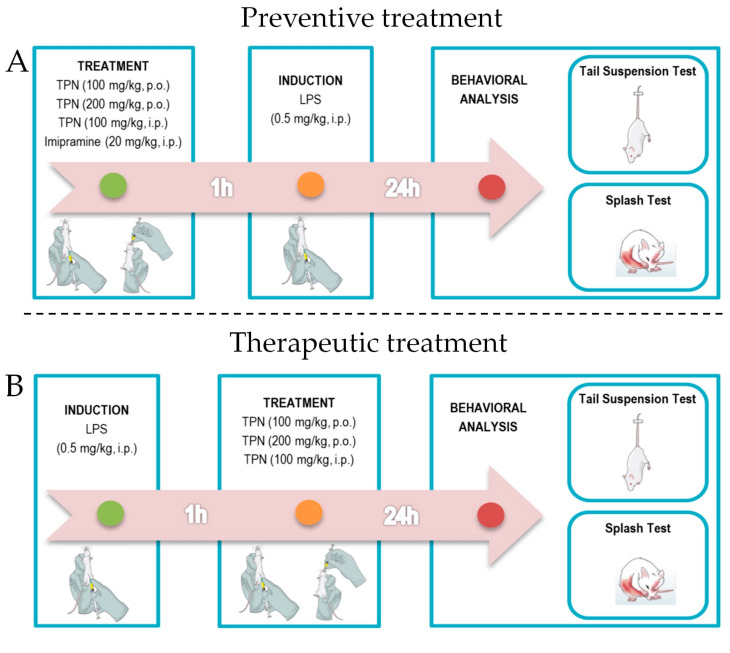

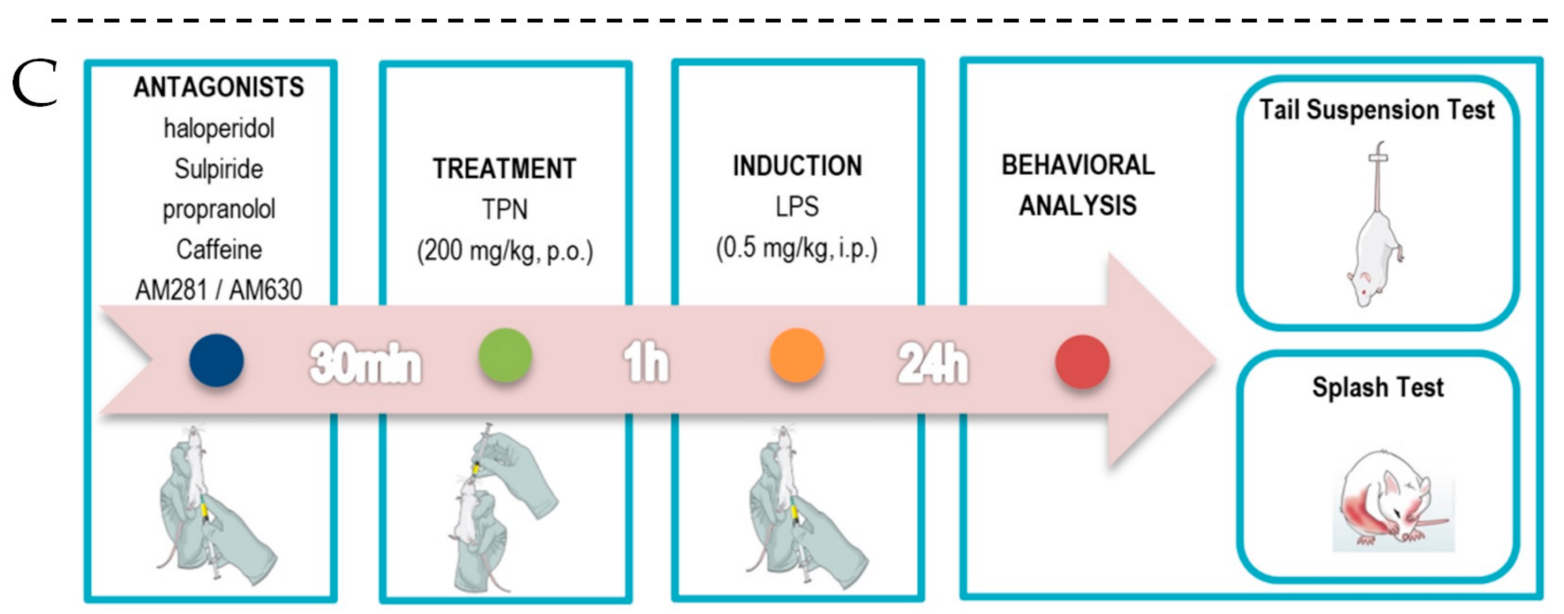
Experimental design. (**A**) For preventive treatment, terpineol (100 mg/kg, p.o. and i.p.; and 200 mg/kg, p.o.) was administered 1 h before lipopolysaccharide (LPS) injection, and we performed the behavioral analysis—tail suspension test (TST) and splash test (ST)—24 h after LPS administration. (**B**) For therapeutic treatment, terpineol (100 mg/kg, p.o. and i.p.; and 200 mg/kg, p.o.) was administrated 1 h after LPS injection, and behavioral analysis was recorded 24 h after the administration of LPS. (**C**) To investigate the mechanisms underlying the antidepressant-like effect of terpineol (200 mg/kg, p.o.), adenosinergic, monoaminergic, and cannabinoid systems were analyzed. Thirty minutes after each treatment administration terpineol (200 mg/kg, p.o.) was given to the animals, and then 1-h later LPS (0.5 mg/kg, i.p.) was administered. Figure created using the Mind the Graph platform.

Three treatments (100 mg/kg, p.o. and i.p.; and 200 mg/kg, p.o.) were used in the tail suspension test (TST) and splash test (ST) to assess the antidepressant-like effect of terpineol. Based on a pilot study (3-4 animals per group), and in previous studies [18,22,39,40,41] doses and administration routes of terpineol were selected to investigate its potential antidepressant-like effect on LPS-induced behavioral alterations. The routes of administration of the treatments were intraperitoneal (i.p.), so that the hepatic first-pass effect was avoided, while the oral route was chosen aiming at translation with use in humans [42]. Imipramine (20 mg/kg, i.p.) was used as a positive control [43,44,45,46]. Terpineol was diluted in saline with 0.5% Tween 80 and administered 1 h before (preventive treatment—Figure 1A) or 1 h after (therapeutic treatment—Figure 1B) of the LPS injection. The control group received saline with 0.5% of Tween 80 as the vehicle. Behavioral analysis was recorded 24 h after the administration of LPS (Figure 1). Adenosinergic, monoaminergic, and cannabinoid systems were analyzed to investigate the mechanisms underlying the antidepressant-like effect of terpineol (200 mg/kg, p.o.). To test A1 or A2 adenosine receptor involvement, the mice were pre-treated with caffeine (3 mg/kg, i.p.) [47]. The mice were pre-treated with haloperidol (0.2 mg/kg, i.p.) [25,38], sulpiride (50 mg/kg, i.p.) [25,38], or propranolol (2 mg/kg i.p.) in order to test the monoaminergic system involvement [48]. The mice were pre-treated with AM281 (1 mg/kg, i.p.) or AM630 (1 mg/kg, i.p.) (Table 1) in order to test CB1 and CB2 cannabinoid receptor involvement [49]. Thirty minutes after each treatment administration terpineol (200 mg/kg, p.o.) was given to the animals, and then 1-h later LPS (0.5 mg/kg, i.p.) was administered (Figure 1C).

### 2.6. Tail Suspension Test (TST)

In this behavioral test, the mice were suspended by the tip of the tail for 6 min using adhesive tape. During this period, the immobility time was recorded [50]. The immobility time is considered to be a depressive-like behavior and antidepressant drugs have been used to reverse this parameter and promote escape-related behavior [51].

### 2.7. Splash Test (ST)

This test consisted of squirting sucrose solution (200 μL, 10%) on the dorsal coat of the animal. Because sucrose at this concentration has a high viscosity, it dirties the mouse fur and induces grooming behavior in the animals. After this procedure, the time spent grooming was recorded for 5 min as an index of self-care and motivational behavior [18,52].

### 2.8. Open Field Test (OFT)

The mice were placed individually in a wooden box (40 × 60 × 50 cm) with the floor divided into 12 squares to investigate locomotion activity and exploratory behavior. The crossing number (number of squares crossed by the animal using all paws) was used to evaluate locomotion activity, whereas the rearing behavior (number of times the mouse stood on its hind legs or engaged in vertical exploratory activity) was used to assess exploratory behavior [53].

### 2.9. Statistical Analysis

Statistical analysis was performed by analysis of variance (ANOVA) while using the Newman–Keuls procedure for multiple comparisons and Bonferroni correction with GraphPad Prism version six and IBM SPSS Statistics 21 (Statistical Package for the Social Sciences, Inc., Chicago, IL, USA). All of the data are presented as the mean ± standard error of the mean (SEM) of six to eight mice per group; *p* values of < 0.05, < 0.01 and < 0.001 were considered statistically significant.

## 3. Results and Discussion

### 3.1. ADMET Analysis

Pharmacokinetic and toxicological parameters, namely human oral bioavailability, human intestinal absorption (HIA), blood-brain barrier (BBB) penetration, Caco-2 cell permeability, Ames mutagenesis, carcinogenicity, hepatotoxicity, and others, were calculated using the ADMET SAR online server. Alpha-terpineol (MW = 154.25 g/mol and LogP = 2.50) showed suitable human oral bioavailability (probability > 0.6857), could penetrate the BBB (probability > 0.9923) and Caco-2 cells (probability > 0.8160), as well as it could be absorbed by the human intestine (probability > 0.9941) (Table 2). Relevantly, terpineol was not shown to be a potential substrate for P-glycoprotein (P-gp) or multidrug resistance protein 1 (MDR1), an important protein of the cell membrane that pumps different foreign compounds, including drugs, out of cells, and possibly alters the expected therapeutic drug concentration. Furthermore, terpineol showed low acute oral toxicity—category IV (which includes compounds with LD50 *>* 5000 mg/kg, according to the criterion of United States Environmental Protection Agency—US EPA)—and it did not show any mutagenic effect concerning the Ames test data, carcinogenicity, or hepatotoxicity (Table 2). 

### 3.2. Molecular Modeling of D2 Dopaminergic Receptor and CB1/CB2 Cannabinoid Receptors by Terpineol

**Figure 2:**
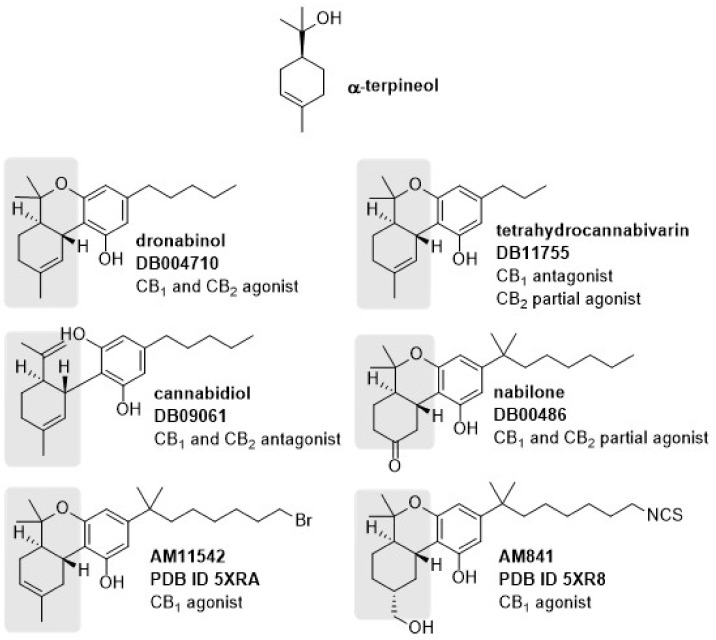
Chemical structures of α-terpineol and cannabinoid receptor ligands. Similar moieties among the structures are highlighted in gray. For each ligand, its DrugBank code and receptor intrinsic activity is given. AM11542 and AM841 are the co-crystallized agonists in Protein Data Bank complexes 5XRA and 5XR8, respectively.

Natural products have been, and continue to be, exceptional sources of bioactive molecules and therapeutically used drugs. Moreover, as a result of their biosynthetic pathways and associated feedback mechanisms, natural products, and their precursors can interact with a range of proteins, including receptors or enzymes [54]. In the era of new technologies, computer-based approaches have allowed the research community to explore computational methods for predicting compound-protein interactions [55]. Ligand-protein docking has been used to predict the predominant binding mode(s) between a ligand and protein of known three-dimensional (3D) structure, and each ligand orientation in the active site of the protein is ranked via a scoring function [56]. Thus, in the drug development processes, in silico assays receive considerable attention for allowing rational drug design, and new initiatives towards better predictions are being continuously undertaken. We performed molecular modeling studies on targets that are likely to predict the antidepressant effect of this compound to study the molecular basis for the terpineol effects. We focused on evaluating possible binding modes of α-terpineol, the major component of the terpineol mixture employed in the experiments reported here (α-terpineol, ~73%). Three-dimensional structures for dopaminergic D2 (6CM4) and CB1/CB2 receptors were available in the PDB. These include four CB1 structures: two in complexes with agonists (5XRA and 5XR8) and two with antagonists (5U09 and 5TGZ) and one antagonist bound CB2 receptor (5ZTY). An analysis of crystallographic ligands from 5XRA and 5XR8, and a search of known ligands for these receptors in the DrugBank database, revealed that terpineol mimics the cyclohexenyl portion of some cannabinoid receptor ligands containing a tetrahydrocannabinol (THC) terpenoid ring system, most of them with some agonist activity (Figure 2).

**Figure 3:**
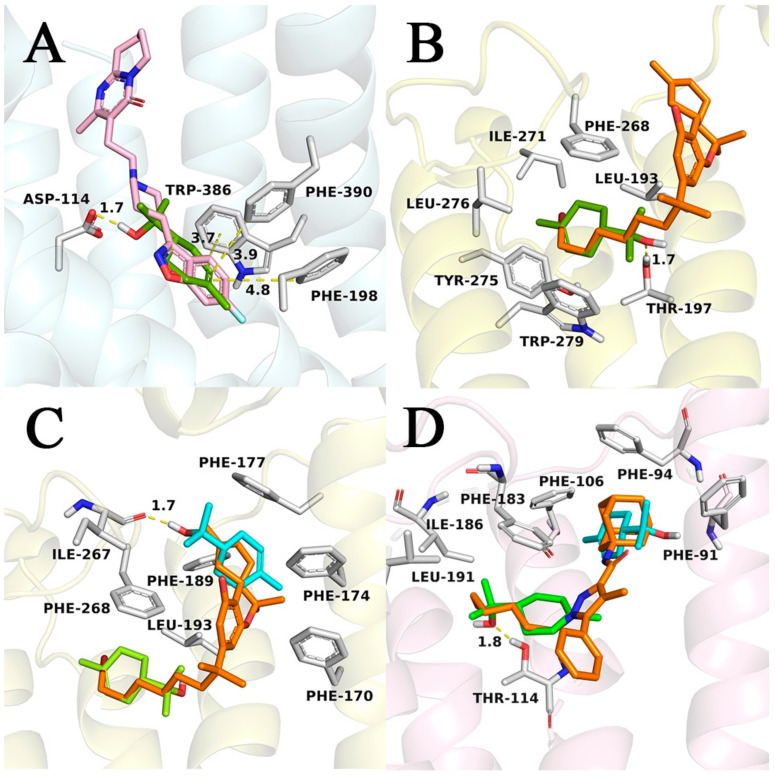
Highest score-predicted binding modes for terpineol against receptors D2, CB1, and CB2. (**A**) Terpineol (green) on the binding pocket of the D2 receptor (blue cartoon). Crystallographic ligand risperidone showed in pink. (**B**) Terpineol (green) on the binding pocket of cannabinoid receptor CB1 (yellow cartoon). Crystallographic ligand AM11542 shown in orange. (**C**) Highest score-predicted binding mode for the second molecule of terpineol (blue) on the binding pocket of cannabinoid receptor CB1 (yellow cartoon). Previously docked terpineol is shown in green and crystallographic ligand AM11542 in orange. (**D**) Highest score-predicted binding mode for first (green) and second (cyan) terpineol in the CB2 receptor binding pocket (pink cartoon). Crystallographic ligand AM10257 is shown in orange for comparison. Hydrogen bond and π-stacking contacts are displayed as yellow dashes and distances (between hydrogen and the hydrogen bond acceptor) are shown in angstroms. Selected residues of the active site are highlighted as white sticks.

We performed docking studies to investigate the binding mode of α-terpineol and predicted ligand-protein interactions. First, redocking studies with crystallographic ligands were performed to validate a docking protocol and choose scoring functions. Studies using multiple scoring functions ultimately led to the choice of Goldscore, with rescoring using Chemscore, as this protocol reproduced crystallographic conformations with RMSDs lower than 1.5 Å. A comparison of the orthosteric binding site of the receptors revealed that the D2 receptor has polar residues, such as Asp, Thr, and Ser, whereas, in cannabinoid receptors, there is a prevalence of hydrophobic residues, such as Phe, Ile, and Leu (Figure 3). The highest score-predicted binding mode of terpineol against the D2 receptor is in the same region occupied by the benzisoxazole ring on the crystallographic ligand risperidone and is predicted to be a hydrogen bond to the Asp114 side chain (distance = 1.7 Å). Conversely, the benzisoxazole ring is capable of multiple π-stacking interactions, which are not possible with terpineol (Figure 3A). A crystallographic structure in a complex with an agonist was used (5XRA), owing to terpineol’s greater similarity with CB1 agonists. The predicted binding modes against the CB1 receptor are consistently positioned deep in the binding site, in a channel formed by helices III, V, and VI, and occupied by the alkyl chain of the crystallographic ligand AM11542, showing high carbon superposition with the latter (Figure 3B). This channel is mainly constituted of hydrophobic residues, such as Leu193, Phe268, Ile271, Tyr275, Leu276, and Trp279, as commonly observed in lipid-binding receptors [33]. In the highest-scoring pose, terpineol is predicted to make a hydrogen bond to the Thr197 side-chain hydroxyl (distance = 1.7 Å). We evaluated whether two ligand molecules could be accommodated at the same time when considering the orthosteric binding site volume and the relatively small size of terpineol. To consider this hypothesis a new docking protocol was run, utilizing as receptor the best solution found in the previous docking, with the predicted pose of terpineol as part of the macromolecule. The results indicated binding poses in which the terpineol was positioned in the region occupied by the cyclohexenyl motif of the crystallographic agonist ligand, differing by a ring flip that allows for the formation of a hydrogen bond to the Ile267 main chain carbonyl (distance = 1.7 Å) on the CB1 receptor (Figure 3C). Finally, we docked terpineol to the CB2 receptor. The predicted binding mode for this receptor occupies a similar region to the observed for CB1, despite terpineol’s flipped orientation and establishment of a hydrogen bond with Thr114 (Figure 3D). The similarity observed is reasonable when considering that CB1 and CB2 receptors have high sequence identity and high similarity at the binding pocket. The main differences are substitutions Phe108/Tyr25, Leu193/Ile110, Ile267/Leu182, and Leu359/Val261, which do not affect the hydrophobic character of the pockets. As for the additional terpineol, docking solutions also occupy a similar region observed for CB1, partially overlapping the adamantyl substituent in the crystallographic antagonist, but not engaging in hydrogen bonds, as the predicted pose was shifted towards the Phe106 residue (Figure 3D). Interestingly, in both cases, terpineol was not positioned near the region that was occupied by the phenyl ring of the crystallographic antagonist, which is believed to have an important role stabilizing the receptor in its inactive form [34]. 

Drug discovery heavily depends on bioinformatics, including the application of molecular docking to hit identification and lead optimization. This technique has accelerated drug discovery regarding reducing time and the cost of the process [57]. Moreover, docking studies make it possible to evaluate a very large library of compounds to identify structures that are most likely bound to a protein receptor [58]. Our molecular modeling studies indicated high similarity between the cannabinoid receptors at the hydrophobic binding site, with the presence of many phenylalanine residues; such a site seems to be compatible with terpineol binding. It is important to mention that the available CB1 crystallographic complexes could be clustered into two different conformations: active, in a complex with an agonist; and inactive, stabilized by an antagonist. Differences in conformation have been discussed already in the literature [33]. Binding, as in the 5XRA complex, induces conformational changes in the structure, most notably inward shifts of helices I and II with the rotation of the side chains of Phe170 and Phe174. Therefore, the binding pocket becomes smaller than in the inactive antagonist-stabilized conformation. Additionally, it is believed that these conformational changes trigger the activation and signaling that are associated with the receptor [33]. Based on the principle that similar structures tend to have the same mechanism of interaction, docking studies were carried out using the PDB structures in the active, constricted conformation, when available. Docking with cannabinoid receptors predicted terpineol poses that superimpose the aliphatic chain of crystallographic ligands. It is well documented that the C-3 alkyl chain of THC analogs plays a significant role in cannabinoid receptor affinity and also selectivity. Substitution for bulky groups is well-tolerated, demonstrating favorable hydrophobic interactions [59]. This region is characterized by the presence of numerous phenylalanine and other hydrophobic residues, and also for not being solvent accessible, which would make terpineol binding entropically favorable via the hydrophobic effect. Based on the assumption that the second molecule of terpineol could bind to the orthosteric binding site of these receptors, docking results showed a new favorable binding region comprised of residues that interact with the cyclohexenyl motif—which is similar to terpineol—in the crystallographic ligands. On the other hand, docking terpineol to the D2 receptor suggests an important role for the hydroxyl, anchoring the molecule near the benzisoxazole portion of the crystallographic ligand. In the crystallographic complex, this pocket is responsible for numerous π-stacking contacts, which cannot be formed with terpineol due to the lack of aromatic motifs in its structure. Additionally, none of the known D2 receptor ligands share structural similarity with terpineol, which suggests that the D2 receptor is less likely to be targeted by this ligand. 

### 3.3. Evaluation of Terpineol’s Antidepressant-Like Effect in the TST, ST, and OFT

**Figure 4:**
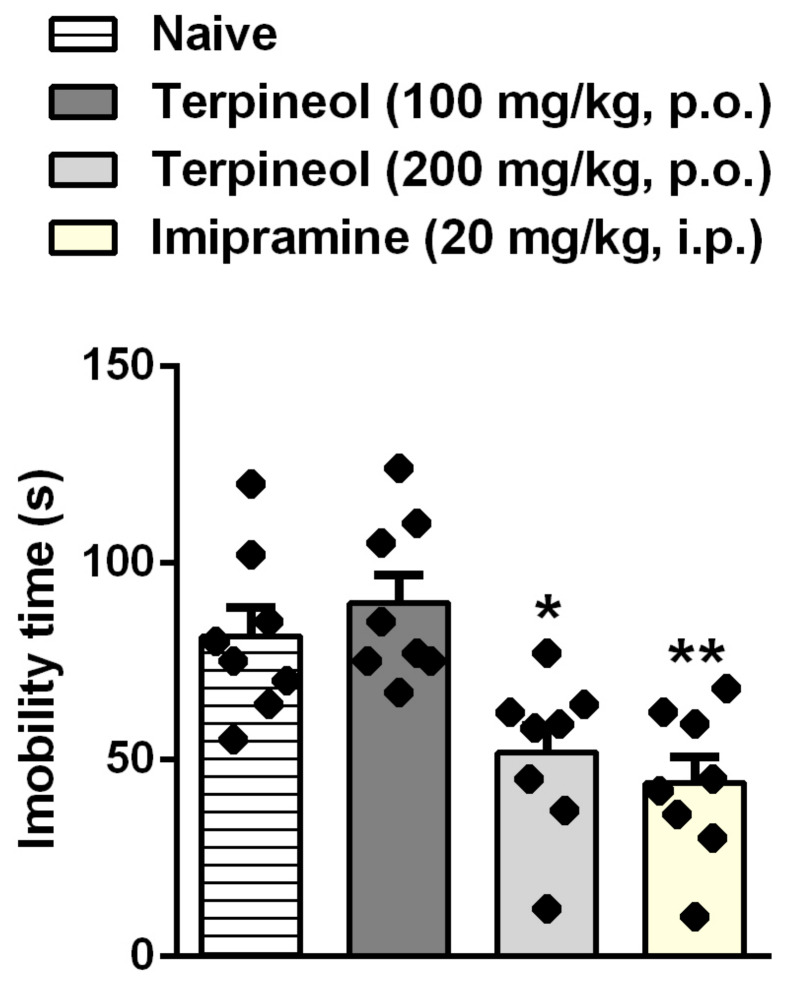
Effect of the treatment with terpineol given orally on the immobility time in the TST. Each column represents the mean ± SEM of eight mice/group, with black dot showing individual mice. Differences between groups are indicated: *p < 0.05 and **p < 0.001 compared with the naive-untreated group.

Figure 4 shows the effects of the terpineol in the immobility time in the TST. The one-way ANOVA showed a significant effect of terpineol treatment on the immobility time in the TST (ANOVA treatment effect: F_3,28_ = 9.706, *p* < 0.05; Figure 4). Terpineol significantly reduced the immobility time in TST at the dose of 200 mg/kg as compared to the control group (Figure 4). Imipramine (20 mg/kg, i.p.), a classic antidepressant drug that is used as the positive control, showed a significant antidepressant effect compared to the control group (Figure 4; *p* < 0.001) in our experimental conditions.

**Figure 5:**
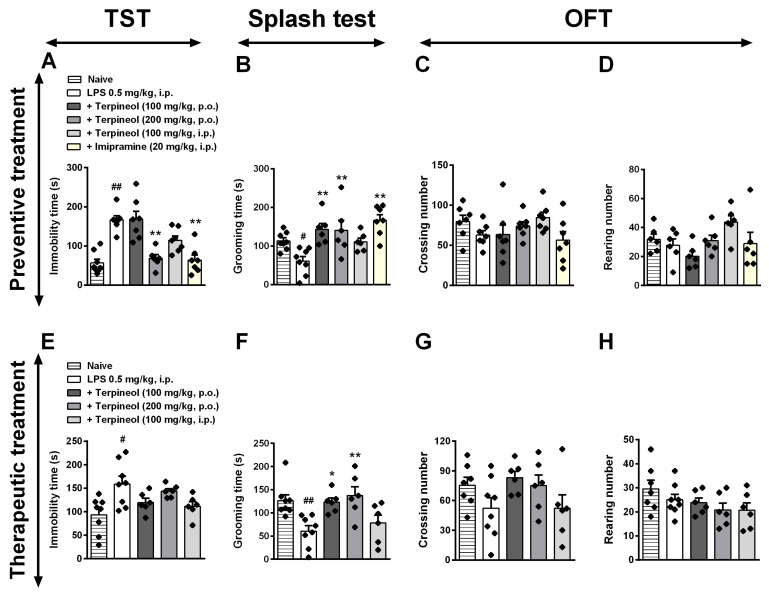
Effect of preventive and therapeutic treatment with terpineol in LPS-induced depressive-like behavior in mice in the tail suspension test (TST: **A**,**E**), splash test (ST: **B**,**F**), and open field test (OFT: **C**,**D** and **G**,**H**). Terpineol (100 mg/kg, p.o.; 200 mg/kg, p.o.; 100 mg/kg, i.p.) was administered 1 h before LPS (0.5 mg/kg, i.p.; preventive treatment—top panel) or 24 h after LPS administration (therapeutic treatment—bottom panel). Imipramine (20 mg/kg, i.p.) was used as a positive control. Data are presented as the mean ± SEM of six to eight mice/group, with black dot showing individual mice. Differences between groups are indicated: *p < 0.05 and **p < 0.01 compared to the LPS group; # *p* < 0.05 and ## *p* < 0.01 compared to the naïve group.

Next, based on the inflammation hypothesis of depression, in this set of experiments, we treated Swiss mice with LPS (0, 5 mg/kg, i.p.) to induce depressive-like behavior. The results show that terpineol (200 mg/kg, p.o.) administered 1 h before LPS significantly decreased the immobility time (ANOVA treatment effect: F_5,29_ = 10.61, *p* < 0.001; Figure 5A) in comparison to the LPS group in the TST. Additionally, terpineol (100 and 200 mg/kg, p.o.) administered 1 h before LPS significantly increased grooming time (ANOVA treatment effect: F_5,29_ = 9.220, *p* < 0.001; Figure 5B) in comparison to the LPS group in the ST. The classic antidepressant imipramine (20 mg/kg, i.p.) was used as the positive control in the TST (Figure 5A; *p* < 0.001) and SP (Figure 5B; *p* < 0.001) and it had a significant effect when compared to the LPS-untreated group. Remarkably, terpineol administered 1 h after LPS failed to inhibit the depressive-like effect induced by LPS administration in the TST (Figure 5E), although terpineol at the oral doses of 100 mg/kg and 200 mg/kg increased the grooming time (ANOVA treatment effect: F_4,24_ = 10.68, *p* < 0.05 and *p* < 0.001, respectively; Figure 5F) as compared to the LPS group in the ST. None of the doses of terpineol used in both protocols (preventive and therapeutic treatment) were able to cause any change in the OFT (Figure 5C–D and G–H), confirming that terpineol does not alter the locomotion pattern when administered 1 h after LPS. In the development of new drugs to control diseases, often a drug that works well in animals is considered not to be effective in humans, because the drug dose cannot be easily translated from one animal species to another. Importantly, the animal dose should not be extrapolated to a human equivalent dose (HED) by a simple conversion that is based on body weight, and the Food and Drug Administration (FDA) has suggested that extrapolation of an animal dose to a human dose can only be performed correctly by normalization to body surface area (BSA), which often is represented in mg/m^2^ [42]. Thus, the human equivalent dose (HED) can be more appropriately calculated by using the formula: HED (mg/kg) = animal dose (mg/kg) × animal *K_m_* / human *K_m_*; with mouse *K_m_* = 3 and human adult *K_m_* = 37, this calculation results in HED values of 486 and 972.60 mg/day for 8.10 and 16.21 mg/kg terpineol, respectively, in a 60-kg person, suggesting that these concentrations may be provided through a daily oral supplement. Additionally, data obtained from Santa Cruz Biotechnology catalog—sc-291877 (Santa Cruz, CA, USA) showed that the calculated photodegradation half-lives for the terpenoid alcohols and esters are in the range from 1.07 to 9.08 h, and calculated half-lives for alpha-terpineol is 1.24 h. Nonetheless, there are no data available on the half-life of terpineol when administered orally, and therefore should be further investigated.

It is known that terpenes present anti-inflammatory effects and they have effect as a monoamine oxidase (MAO) inhibitor [60,61]. Since the monoamines are metabolized by MAO, the inhibition of the enzyme might increase their brain concentrations and, thus, reduce disease symptoms. It could explain why the preventive treatment with terpineol showed antidepressant effect, while the therapeutic treatment failed. Moreover, linalool and limonene, plant-derived monoterpenes alcohol, showed antidepressant activity [62,63]. Linalool (acyclic monoterpene alcohol) and limonene (unsaturated cyclic monoterpenes) both serve as precursors for α-terpineol formation, although linalool seems to be more reactive substrate than limonene, since the protonation in linalool was faster than in limonene [64]. Interestingly, limonene restored the chronic unpredictable mild stress-induced (CUMS) hyperactivity of the hypothalamic-pituitary-adrenal axis and modulated monoamine neurotransmitter levels. Additionally, long-term limonene inhalation reversed CUMS-induced decreased BNDF and TrkB expression in the hippocampus as well as restored the levels of DA, 5-HT, and NE in the hippocampus and prefrontal cortex [63]. Previous evidence has shown terpineol involvement in different pathways that mitigate inflammatory and nociceptive processes, for instance, the inhibition of pro-inflammatory mediators (IL-1β, IL-6, and TNF), COX-2, iNOS, and activation of NF-κB, as well as terpineol upregulated IL-10 expression—a pro-resolution cytokine—during the inhibition of bacterial growth [19,65]. Additionally, 4-terpineol and α-terpineol inhibited LPS-induced pulmonary fibrosis through the modulation of IFN-γ and TGF-β1/SMAD pathway [66], suppressed macrophages proliferation and inhibited NF-κB, p38 or ERK MAPK pathways [21]. Regarding the terpineol effect in nociceptive processes, it was observed that α-terpineol inhibited neuropathic pain by the modulation of the microglial cells and reduction of inflammatory cytokine levels in the spinal cord of rats [67]. A very interesting study conducted by Safaripour and co-authors showed that the antinociceptive effect of α-terpineol seems to be mediated by the l-arginine/SNAP/NO/cGMP/KATP channel pathway [26]. Another study demonstrated that pre-systemic treatment with terpineol inhibited mechanical hyperalgesia induced by carrageenan, TNF, prostaglandin E₂, and dopamine associated with the inhibition of neutrophil influx and nitrite production [41]. Gouveia and colleagues demonstrated that α-terpineol attenuated mechanical hyperalgesia and spontaneous-nociception on cancer pain induced by sarcoma in mice, as well as terpineol increased the tissue antioxidant capacity and reduced inducible nitric oxide synthase immunocontent in the tumor [65]. Additionally, 4-terpineol showed in vitro and in vivo anticancer effects in Hep-G2 hepatocellular carcinoma cells by suppressing cell migration and inducing apoptosis and sub-G1 cell cycle arrest [68] and α-terpineol inhibited iNOS modulating oxidative stress in cancer pain [65]. These shreds of evidence support terpineol involvement in different pathways of inflammatory processes that could be shared with depression development, since it is a multifactorial disorder that includes an inflammatory component. Recent evidence demonstrates that terpenoids are small, fat-soluble organic molecules that can transfer across nasal mucosa if inhaled or penetrate through the skin after topical application, enter into the blood, and, particularly, cross the blood-brain barrier [69,70], where they modulate brain function [71]. From this, it is possible to suggest that terpineol (Table 2), as well as linalool and limonene, may cross the blood-brain barrier during LPS-induced depressive behavior and modulate some essential aspects that are related to its development, although further in vivo experiments are needed to confirm this hypothesis.

### 3.4. Antidepressant-Like Effect of Terpineol is Not Dependent on the A1 and A2A Adenosine Receptor Signaling Pathway

**Figure 6:**
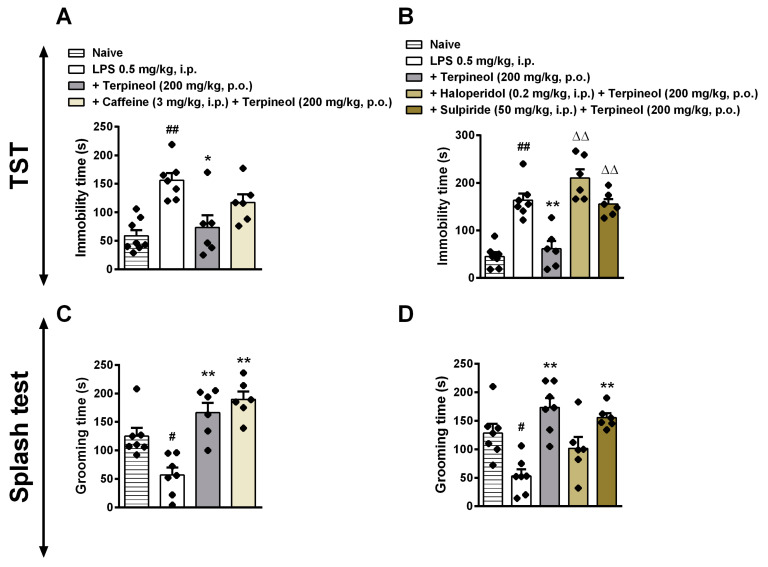
Involvement of A1 and A2 adenosine receptors and D1 and D2 dopamine receptors in the terpineol antidepressant-like effect in the tail suspension test (TST: **A**,**B**) and splash test (ST: **C**,**D**). Mice were treated with caffeine (a nonselective adenosine receptor antagonist: 3 mg/kg, i.p.—left panel), haloperidol (a nonselective dopaminergic receptor antagonist: 0.2 mg/kg, i.p.—right panel) or sulpiride (a selective dopamine D2 receptor antagonist: 50 mg/kg, i.p.—right panel). After 30 min the animals received terpineol (200 mg/kg, p.o.), and 1 h later they were treated with LPS (0.5 mg/kg, i.p.). After 24 h of LPS administration, the behavioral analyses were performed. The data are presented as the mean ± SEM of six to eight mice/group, with black dot showing individual mice. Differences between groups are indicated: *p < 0.05 and **p < 0.01 compared to the LPS group; # *p* < 0.05 and ## *p* < 0.01 compared to the naïve group; ^ΔΔ^*p* < 0.01 compared to the terpineol group.

Adenosine is a pleiotropic bioactive compound with potent neuromodulatory properties. Owing to its ability to easily cross the blood-brain barrier, it can act as a signaling molecule between the periphery and brain environment [72]. Previous results showed that caffeine—a nonselective adenosine A1/A2A antagonist—improved depression and anxiety effects at low doses in normal healthy patients [73]. Additionally, caffeine reversed the swimming deficits promoted by reserpine, suggesting that the effect of adenosine is related to monoaminergic modulation during depression [74]. We investigated the possible relationship between terpineol and adenosine receptors with the aim to identify the mechanisms behind the preventive effect of terpineol on LPS-induced depressive-like behavior. Thus, we demonstrated that pre-treatment with caffeine (3 mg/kg, i.p.) did not alter anti-immobility effect of terpineol in the TST (ANOVA treatment effect: F_3,23_ = 9.76, *p* = 0.1649; Figure 6A) and ST (ANOVA treatment effect: F_3,22_ = 16.02, *p* = 0.8725; Figure 6c). Altogether, it is possible to conclude that different of other terpenoids, such as linalool and limonene, which mediated its analgesic [75] and sedative effects [76] by the activation of adenosine receptors, the beneficial effects of terpineol on depressive-like and motivational behavior induced by LPS appears not to be dependent on the A1 and A2A adenosine receptors.

### 3.5. Involvement of Dopaminergic Receptors in Terpineol’s Antidepressant-Like Effects

It is known that the downregulation of serotonin/noradrenaline/dopamine in the synaptic cleft could be responsible for depressive disorders, as well as the treatment of depression aims for restoring monoamine levels [77,78,79]. Interestingly, recent findings have shown that LPS induced a significant reduction of D3 receptor in areas that are related to the mesolimbic dopaminergic system and that treatment with NGB 2904—a D3 receptor-selective antagonist—induced depressive-like behavior compared with a vehicle-treated control group [80]. Considering that linalool and β-pinene, as well as terpineol, showed an antidepressant-like effect through interaction with the monoaminergic system [81]; next, we investigated whether the blockade of dopaminergic receptors would be able to revert the preventive antidepressant-like effect of terpineol. Interestingly, pre-treatment with haloperidol (0.2 mg/kg, i.p., a nonselective dopaminergic receptor antagonist) or sulpiride (50 mg/kg, i.p., a selective dopamine D2 receptor antagonist) increased the immobility time in the TST (ANOVA treatment effect: F_4,21_ = 18.26, *p* < 0.001; Figure 6B) when compared to the terpineol group, suggesting the involvement of D2 dopaminergic receptors in the action of terpineol. However, this effect was not observed in the ST (*p* > 0.05; Figure 6D). Haloperidol or sulpiride alone treatment did not affect the behavior and immobility time, as previously described [25,82,83,84]. There is a wide variety of essential oils recognized by its antidepressant-like effect [63,64]. In this way, β-pinene demonstrated antidepressant activity through dopaminergic receptors activation, particularly D1 receptors instead of D2 receptors [81]. Curiously, lemon oil rich in α-pinene showed anxiolytic and antidepressant-like effects. Despite, unlike β-pinene and terpineol, these effects were linked to suppression of DA activity instead of its activation [85]. Thus, it is possible to hypothesize that terpineol, as well β-pinene, could interact with dopaminergic receptors (as shown in Figure 3) in order to prevent the depressive like-behavior and loss of self-care, but this interaction still needs to be better investigated.

### 3.6. Role of Cannabinoid Receptor Signaling Pathway in the Antidepressant-Like Effect Caused by Terpineol

**Figure 7:**
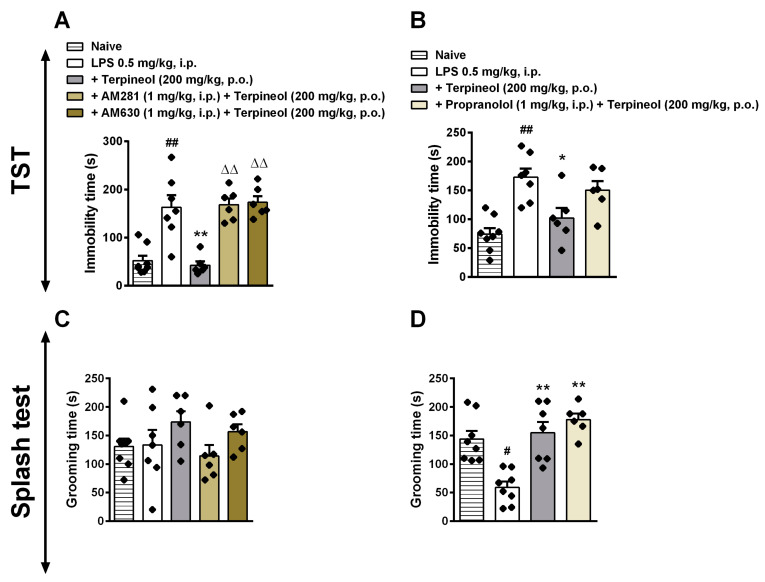
Involvement of CB1 and CB2 cannabinoid receptors and β-adrenergic receptors in the terpineol antidepressant-like effect in the tail suspension test (TST: **A**,**B**) and splash test (ST: **C**,**D**). Mice were treated with a selective CB1 cannabinoid receptor antagonist/inverse agonist (AM281: 1 mg/kg, i.p.—left panel), a potent and selective inverse agonist for the CB2 cannabinoid receptor (AM630: 1 mg/kg, i.p.—left panel) or with the β-adrenoceptor antagonist propranolol (2 mg/kg, i.p.—right panel). After 30 min the animals received terpineol (200 mg/kg, p.o.), and 1 h later they were treated with LPS (0.5 mg/kg, i.p.). After 24 h of LPS administration, the behavioral analyses were performed. The data are presented as the mean ± SEM of six to eight mice/group, with black dot showing individual mice. Differences between groups are indicated: *p < 0.05 and **p < 0.01 compared to the LPS group; # *p* < 0.05 and ## *p* < 0.01 compared to the naïve group; ^ΔΔ^*p* < 0.01 compared to the terpineol group.

Currently, the endocannabinoid system has been recognized as a prominent promoter of emotional homeostasis and, in this way, some studies have suggested that depression coincides with low levels of endocannabinoid activity [86,87,88]. Previous findings showed a general anti-inflammatory role for the CB2 receptor in mice that were exposed to sub-chronic restraint and acoustic stress, suggesting CB2 receptor as a cellular target for the treatment of stress-related disorders that are associated with the neuroinflammatory core, such as depression [89]. CB1 receptor is also involved in depression, with neural stem cell lineage-specific CB1 receptor-regulated neurogenesis and plasticity in the adult mouse hippocampus leading to decreased short-term spatial memory and increased depressive-like behavior [90]. Here, our results also show CB1 and CB2 receptor participation in the antidepressant-like effect of terpineol. Pre-treatment with a selective CB1 cannabinoid receptor antagonist/inverse agonist (AM281, 1 mg/kg, i.p.) or a potent and selective inverse agonist for the CB2 cannabinoid receptor (AM630, 1 mg/kg, i.p.) increased the immobility time in the TST (ANOVA treatment effect: F_4,20_ = 8.482, *p* < 0.01; Figure 7A) as compared to the terpineol group. Taken together, it is possible to suggest that the antidepressant-like effect of terpineol is dependent on cannabinoid receptors. However, this effect was not observed in the ST (*p* > 0.05; Figure 7C). Concerning the mechanism of action, previous evidence demonstrated that essential oils with α-terpineol exert an antidepressant-like effect by dopaminergic [91], serotonergic [91,92,93], noradrenergic [91,92], and cholinergic [94] receptors, but there is a lack of evidence about cannabinoid receptors. It was observed that cannabichromene extract altered behavioral despair on the mouse TST of depression [95]. Furthermore, the phytocannabinoid (cannabigerol) extract showed altered behavioral despair during the animal model of depression [96]. Later, the cannabigerol effect was described as activating α2-adrenoceptors, binding to CB1 and CB2 cannabinoid receptors, and blocking CB1 and serotonin 1A (5-HT1A) receptors [97]. The data obtained herein show, while using pharmacological and molecular approaches, that both CB1 and CB2 receptors are involved with the antidepressant-like effect of terpineol, suggesting its cannabimimetic action.

### 3.7. Involvement of β-Adrenoceptor in Terpineol’s Antidepressant-Like Effects

A possible risk for the development of depression could lay in the combination of stress-induced transient activation of α1-adrenoceptors and the activation of the hypothalamic-pituitary-adrenal (HPA) axis, which would lead to higher activation of the *locus coeruleus* [98,99]. It has been observed that downregulation of β1-, β3-, and α2-adrenoceptors might be involved in the antidepressant effects [100]. Herein, we evaluated whether the terpineol antidepressant-like effect would be affected by β-adrenoceptor antagonist propranolol (2 mg/kg i.p.). Propranolol treatment did not significantly change the antidepressant-like effect of terpineol after LPS administration, suggesting that β-adrenergic receptors are not involved in this effect, as shown in Figure 7B,D.

## 4. Conclusions

**Figure 8:**
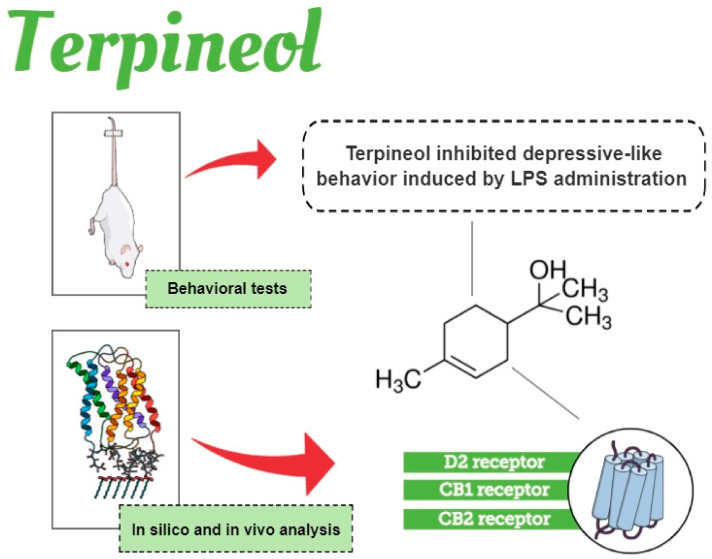
Schematic diagram illustrating the beneficial effects of terpineol during LPS-induced depressive behavior. Terpineol is monoterpene alcohol present in the essential oils of several species of plants, such as *Eucalyptus, Artemisia princeps Pamp,* and *Cannabis sativa*. Briefly, herein we demonstrated through in silico analysis and a behavioral assay that terpineol inhibited depressive-like behavior induced by lipopolysaccharide (LPS) injection, possibly through modulation of dopamine receptor type 2 (D2R), cannabinoid receptor type 1 (CB1R), and cannabinoid receptor type 2 (CB2R). Infographic created using the Mind the Graph platform (https://www.mindthegraph.com).

In summary, when considering the structures studied, we speculate that cannabinoid receptors CB1 and CB2 are the most promising targets and should be prioritized in further studies regarding the mechanism of action of terpineol, possibly acting as an agonist (see proposed scheme in Figure 8), although additional studies are necessary to test this hypothesis.

## Figures and Tables

**Table 1:** Experimental protocols and the respective doses used.

Drugs	Dose	References
Haloperidol—nonselective dopaminergic receptor antagonist	0.2 mg/kg (i.p.)	[25,38]
Sulpiride—selective dopamine D2 receptor antagonist	50 mg/kg (i.p.)	[25,38]
Propranolol—β-adrenoceptor antagonist	2 mg/kg (i.p.)	[48]
Caffeine—nonselective adenosine receptor antagonist	3 mg/kg (i.p.)	[47]
AM281—selective CB1 receptor antagonist / inverse agonist	1 mg/kg (i.p.)	[49]
AM630—selective inverse agonist for the CB2 receptor	1 mg/kg (i.p.)	[49]

**Table 2:** Absorption, distribution, metabolism, excretion, and toxicity profile (ADMET) predicted profile of the α-terpineol.

Model	Result	Probability
Absorption		
Blood-Brain Barrier	BBB+	0.9923
Human intestinal absorption	HIA+	0.9941
Caco-2 permeability	Caco2+	0.8160
Human oral bioavailability	+	0.6857
P-glycoprotein substrate	Non-substrate	0.9405
P-glycoprotein inhibitor	Non-inhibitor	0.9843
Renal organic cation transporter	Non-inhibitor	0.8024
**Distribution**		
Subcellular localization	Lysosomes	0.4268
**Metabolism**		
OATP1B1 inhibitor	Inhibitor	0.9677
OATP2B1 inhibitor	Non-inhibitor	0.8466
OATP1B3 inhibitor	Inhibitor	0.8719
MATE1 inhibitor	Non-inhibitor	0.9600
OCT2 inhibitor	Non-inhibitor	0.6750
BSEP inhibitor	Non-inhibitor	0.9059
CYP2D6 substrate	Non-substrate	0.7630
CYP3A4 inhibition	Non-inhibitor	0.8411
CYP2C19 inhibition	Non-inhibitor	0.7049
CYP2D6 inhibition	Non-inhibitor	0.9322
UGT catalyzed	+	0.7000
**Toxicity**		
Ames mutagenesis	Non-AMES toxic	0.9800
Carcinogens	Non-carcinogens	0.8429
Acute oral toxicity	IV	0.6381
Carcinogenicity (trinary)	Non-required	0.5811
Hepatotoxicity	Non-toxic	0.7750
Eye corrosion	Non-toxic	0.8463
Biodegradation	+	0.7750
**ADMET Predicted Profile–Regression**		
Water solubility	−2.336 LogS	-
Plasma protein binding	0.652 (100%)	-
Acute oral toxicity	2.137 kg/mol	-
Fish aquatic toxicity	0.6781 pLC50, mg/L	-
Tetrahymena pyriformis	−0.468 pIGC50 (μg/L)	-
Rat acute toxicity	1.5063 LD50, mol/kg	-

OAT: organic anion transporting polypeptide; BSEP: bile salt export pump; CYP: cytochromes P450; UGT: uridine 5’–diphospho–glucuronosyltransferase; LC50: lethal concentration; LD50: lethal dose.

## References

[1] Malhi G.S., Mann J.J. (2018). Depression. Lancet.

[2] McLaughlin K.A. (2011). The Public Health Impact of Major Depression: A Call for Interdisciplinary Prevention Efforts. Prev. Sci..

[3] Kessler R.C. (2011). The Costs of Depression. Psychiatr. Clin. North. Am..

[4] Irwin M.R. (2002). Psychoneuroimmunology of Depression: Clinical Implications. Brain, Behav. Immun..

[5] Gold S.M., Irwin M.R. (2006). Depression and Immunity: Inflammation and Depressive Symptoms in Multiple Sclerosis. Neurol. Clin..

[6] Hillhouse T.M., Porter J.H. (2015). A brief history of the development of antidepressant drugs: From monoamines to glutamate. Exp. Clin. Psychopharmacol..

[7] Blackburn T.P. (2019). Depressive disorders: Treatment failures and poor prognosis over the last 50 years. Pharmacol. Res. Perspect..

[8] Miller A.H., Raison C.L. (2015). The role of inflammation in depression: From evolutionary imperative to modern treatment target. Nat. Rev. Immunol..

[9] Tzavara E.T., Davis R.J., Perry K.W., Li X., Salhoff C., Bymaster F.P., Witkin J.M., Nomikos G.G. (2003). The CB1 receptor antagonist SR141716A selectively increases monoaminergic neurotransmission in the medial prefrontal cortex: Implications for therapeutic actions. Br. J. Pharmacol..

[10] Ming Z., Sawicki G., Bekar L.K. (2015). Acute systemic LPS-mediated inflammation induces lasting changes in mouse cortical neuromodulation and behavior. Neurosci. Lett..

[11] Casaril A.M., Domingues M., Fronza M., Vieira B., Begnini K., Lenardão E.J., Seixas F., Collares T., Nogueira C.W., Savegnago L. (2017). Antidepressant-like effect of a new selenium-containing compound is accompanied by a reduction of neuroinflammation and oxidative stress in lipopolysaccharide-challenged mice. J. Psychopharmacol..

[12] O’Connor J., Lawson M., André C., Moreau M., Lestage J., Castanon N., Kelley K.W., Dantzer R. (2008). Lipopolysaccharide-induced depressive-like behavior is mediated by indoleamine 2,3-dioxygenase activation in mice. Mol. Psychiatry.

[13] Ji M., Mao M., Li S., Zhang L., Qiu L., Li B., Xia J., Yang J. (2019). Acute ketamine administration attenuates lipopolysaccharide-induced depressive-like behavior by reversing abnormal regional homogeneity in the nucleus accumbens. NeuroReport.

[14] Cordeiro R.C., Filho A.J.M.C., Gomes N.S., Tomaz V.D.S., Medeiros C.D., Queiroz A.I.D.G., Maes M., Macedo D.S., Carvalho A.F. (2019). Leptin Prevents Lipopolysaccharide-Induced Depressive-Like Behaviors in Mice: Involvement of Dopamine Receptors. Front. Psychol..

[15] Taniguti E., Ferreira Y., Stupp I., Fraga-Junior E., Doneda D., Lopes L., Rios-Santos F., Lima E., Buss Z., Viola G. (2019). Atorvastatin prevents lipopolysaccharide-induced depressive-like behaviour in mice. Brain Res. Bull..

[16] Remus J.L., Dantzer R. (2016). Inflammation Models of Depression in Rodents: Relevance to Psychotropic Drug Discovery. Int. J. Neuropsychopharmacol..

[17] Habtemariam S. (2017). Antidiabetic Potential of Monoterpenes: A Case of Small Molecules Punching above Their Weight. Int. J. Mol. Sci..

[18] Quintans-Júnior L.J., Oliveira M.G., De Santana M.F., Santana M.T., Guimarães A., Siqueira J.S., De Sousa D.P., Almeida R.N. (2011). α-Terpineol reduces nociceptive behavior in mice. Pharm. Boil..

[19] Trinh H.-T., Lee I.-A., Hyun Y.-J., Kim N.-H. (2011). Artemisia princeps Pamp. Essential oil and its constituents eucalyptol and α-terpineol ameliorate bacterial vaginosis and vulvovaginal candidiasis in mice by inhibiting bacterial growth and NF-κB activation. Planta Medica.

[20] Russo E.B., Marcu J. (2017). Cannabis Pharmacology: The Usual Suspects and a Few Promising Leads. The Roles of Vasopressin and Oxytocin in Memory Processing.

[21] Nogueira M.N.M., De Aquino S.G., Junior C.R., Spolidorio D.M.P. (2014). Terpinen-4-ol and alpha-terpineol (tea tree oil components) inhibit the production of IL-1β, IL-6 and IL-10 on human macrophages. Inflamm. Res..

[22] Oliveira M.G., De Brito R.G., Santos P.L., Filho H.G.D.A., Quintans-Júnior L.J., Menezes P.P., Serafini M.R., Carvalho Y., Silva J.C., Almeida J.R.G.D.S. (2016). α-Terpineol, a monoterpene alcohol, complexed with β-cyclodextrin exerts antihyperalgesic effect in animal model for fibromyalgia aided with docking study. Chem. Interactions.

[23] Parvardeh S., Moghimi M., Eslami P., Masoudi A. (2016). α-Terpineol attenuates morphine-induced physical dependence and tolerance in mice: Role of nitric oxide. Iran. J. Basic Med. Sci.

[24] Ferber S.G., Namdar D., Hen-Shoval D., Eger G., Koltai H., Shoval G., Shbiro L., Weller A. (2020). The “Entourage Effect”: Terpenes Coupled with Cannabinoids for the Treatment of Mood Disorders and Anxiety Disorders. Curr. Neuropharmacol..

[25] Binfaré R.W., Mantovani M., Budni J., Dos Santos A.R.S., Rodrigues A.L.S. (2010). Involvement of dopamine receptors in the antidepressant-like effect of melatonin in the tail suspension test. Eur. J. Pharmacol..

[26] Safaripour S., Nemati Y., Parvardeh S., Ghafghazi S., Fouladzadeh A., Moghimi M. (2018). Role of l -arginine/SNAP/NO/cGMP/KATP channel signalling pathway in antinociceptive effect of α-terpineol in mice. J. Pharm. Pharmacol..

[27] Cosenza M., Gifford A.N., Gatley S.J., Pyatt B., Liu Q., Makriyannis A., Volkow N.D. (2000). Locomotor activity and occupancy of brain cannabinoid CB1 receptors by the antagonist/inverse agonist AM281. Synap..

[28] Paszcuk A.F., Dutra R.C., Da Silva K.A.B.S., Quintão N.L.M., Campos M.M., Calixto J.B. (2011). Cannabinoid Agonists Inhibit Neuropathic Pain Induced by Brachial Plexus Avulsion in Mice by Affecting Glial Cells and MAP Kinases. PLoS ONE.

[29] Bento A.F., Marcon R., Dutra R.C., Claudino R.F., Cola M., Leite D.F., Calixto J.B. (2011). Beta-Caryophyllene inhibits dextran sulfate sodium-induced colitis in mice through CB2 receptor activation and PPARgamma pathway. Am. J. Pathol..

[30] Cheng F., Li W., Zhou Y., Shen J., Wu Z., Liu G., Lee P.W., Tang Y. (2012). admetSAR: A Comprehensive Source and Free Tool for Assessment of Chemical ADMET Properties. J. Chem. Inf. Model..

[31] Morris G.M., Lim-Wilby M. (2008). Molecular Docking. Advanced Structural Safety Studies.

[32] Wang S., Che T., Levit A., Shoichet B.K., Wacker D., Roth B.L. (2018). Structure of the D2 dopamine receptor bound to the atypical antipsychotic drug risperidone. Nature.

[33] Hua T., Vemuri K., Nikas S.P., LaPrairie R.B., Wu Y., Qu L., Pu M., Korde A., Jiang S., Ho J.-H. (2017). Crystal structures of agonist-bound human cannabinoid receptor CB1. Natature.

[34] Li X., Hua T., Vemuri K., Ho J.-H., Wu Y., Wu L., Popov P., Benchama O., Zvonok N., Locke K. (2019). Crystal Structure of the Human Cannabinoid Receptor CB2. Cell.

[35] Jones G., Willett P., Glen R.C., Leach A., Taylor R. (1997). Development and validation of a genetic algorithm for flexible docking 1 1Edited by F.E. Cohen. J. Mol. Biol..

[36] Kilkenny C., Browne W.J., Cuthill I.C., Emerson M., Altman U.G. (2010). Improving bioscience research reporting: The ARRIVE guidelines for reporting animal research. PLoS Boil..

[37] McGrath J., Lilley E. (2015). Implementing guidelines on reporting research using animals (ARRIVE etc.): New requirements for publication in BJP. Br. J. Pharmacol..

[38] Lieberknecht V., Cunha M., Junqueira S.C., Coelho I.D.S., De Souza L., Dos Santos A.R.S., Rodrigues A.L.S., Dutra R.C., Dafre A.L. (2017). Antidepressant-like effect of pramipexole in an inflammatory model of depression. Behav. Brain Res..

[39] Pina L.T.S., Ferro J.N.S., Rabelo T.K., Oliveira M.A., Scotti L., Scotti M.T., Walker C.I.B., Barreto E.O., Júnior L.J.Q., Guimarães A. (2018). Alcoholic monoterpenes found in essential oil of aromatic spices reduce allergic inflammation by the modulation of inflammatory cytokines. Nat. Prod. Res..

[40] Choi Y.-J., Sim W.-C., Choi H.K., Lee S., Lee B.-H. (2013). α-Terpineol induces fatty liver in mice mediated by the AMP-activated kinase and sterol response element binding protein pathway. Food Chem. Toxicol..

[41] Oliveira M.G.B., Marques R.B., De Santana M.F., Santos A.B.D., Brito F.A., Barreto E.O., De Sousa D.P., Almeida F.R.C., Badauê-Passos D., Antoniolli A.R. (2012). α-Terpineol Reduces Mechanical Hypernociception and Inflammatory Response. Basic Clin. Pharmacol. Toxicol..

[42] Reagan-Shaw S., Nihal M., Ahmad N. (2007). Dose translation from animal to human studies revisited. FASEB J..

[43] Sharma R.K., Singh T., Mishra A., Goel R.K. (2017). Relative Safety of Different Antidepressants for Treatment of Depression in Chronic Epileptic Animals Associated with Depression. J. Epilepsy Res..

[44] Zomkowski A.D.E., Hammes L., Lin J., Calixto J.B., Dos Santos A.R.S., Rodrigues A.L.S. (2002). Agmatine produces antidepressant-like effects in two models of depression in mice. NeuroReport.

[45] Mantovani M., Pértile R., Calixto J.B., Dos Santos A.R.S., Rodrigues A.L.S. (2003). Melatonin exerts an antidepressant-like effect in the tail suspension test in mice: Evidence for involvement of N-methyl-D-aspartate receptors and the L-arginine-nitric oxide pathway. Neurosci. Lett..

[46] De Rezende V.B., Rosa D.V., Comim C.M., Magno L.A.V., Rodrigues A.L.S., Vidigal P., Jeromin A., Quevedo J., Silva M.A.R. (2014). NCS-1 deficiency causes anxiety and depressive-like behavior with impaired non-aversive memory in mice. Physiol. Behav..

[47] Lobato K.R., Binfaré R.W., Budni J., Da Rosa A., Dos Santos A.R.S., Rodrigues A.L.S. (2008). Involvement of the adenosine A1 and A2A receptors in the antidepressant-like effect of zinc in the forced swimming test. Prog. Neuro-Psychopharmacology Boil. Psychiatry.

[48] Freitas A.E., Budni J., Lobato K.R., Binfaré R.W., Machado D.G., Jacinto J., Veronezi P.O., Pizzolatti M.G., Rodrigues A.L.S. (2010). Antidepressant-like action of the ethanolic extract from Tabebuia avellanedae in mice: Evidence for the involvement of the monoaminergic system. Prog. Neuro-Psychopharmacology Boil. Psychiatry.

[49] Neves L.M.S., Gonçalves E.C.D., Cavalli J., Vieira G., Laurindo L.R., Simões R.R., Coelho I.S., Dos Santos A.R.S., Marcolino A.M., Cola M. (2017). Photobiomodulation Therapy Improves Acute Inflammatory Response in Mice: The Role of Cannabinoid Receptors/ATP-Sensitive K+ Channel/p38-MAPK Signalling Pathway. Mol. Neurobiol..

[50] Yan H.-C., Cao X., Das M., Zhu X.-H., Gao T.-M. (2010). Behavioral animal models of depression. Neurosci. Bull..

[51] Abelaira H.M., Réus G.Z., Quevedo J. (2013). Animal models as tools to study the pathophysiology of depression. Rev. Bras. de Psiquiatr..

[52] Diaz S.L., Narboux-Nême N., Boutourlinsky K., Doly S., Maroteaux L., Information P.E.K.F.C. (2016). Mice lacking the serotonin 5-HT 2B receptor as an animal model of resistance to selective serotonin reuptake inhibitors antidepressants. Eur. Neuropsychopharmacol..

[53] Machado D.G., Cunha M., Neis V.B., Balen G.O., Colla A., Grando J., Brocardo P.S., Bettio L., Capra J.C., Rodrigues A.L.S. (2012). Fluoxetine reverses depressive-like behaviors and increases hippocampal acetylcholinesterase activity induced by olfactory bulbectomy. Pharmacol. Biochem. Behav..

[54] Laraia L., Robke L., Waldmann H. (2018). Bioactive Compound Collections: From Design to Target Identification. Chem.

[55] Hoque S.U., Chowdhury M.S., Paul A., Barua J., Zannat S.S., Hasan M., Das Gupta S., Barua S., Ahmed S., Kabir M.S.H. (2018). In vivo analgesic effect of different extracts of Hopea odorata leaves in mice and in silico molecular docking and ADME/T property analysis of some isolated compounds from this plant. J. Basic Clin. Physiol. Pharmacol..

[56] Meng X.-Y., Zhang H.-X., Mezei M., Cui M. (2011). Molecular docking: A powerful approach for structure-based drug discovery. Curr. Comput. Drug Des..

[57] Singh J., Chuaqui C.E., Boriack-Sjodin P., Lee W.-C., Pontz T., Corbley M.J., Cheung H.-K., Arduini R.M., Mead J.N., Newman M.N. (2003). Successful shape-Based virtual screening: The discovery of a potent inhibitor of the type I TGFβ receptor kinase (TβRI). Bioorganic Med. Chem. Lett..

[58] Rester U. (2008). From virtuality to reality–Virtual screening in lead discovery and lead optimization: A medicinal chemistry perspective. Curr. Opin. drug Discov. Dev..

[59] Bow E.W., Rimoldi J. (2016). The Structure–Function Relationships of Classical Cannabinoids: CB1/CB2 Modulation. Perspect. Med. Chem..

[60] Herraiz T., Guillén H. (2018). Monoamine Oxidase-A Inhibition and Associated Antioxidant Activity in Plant Extracts with Potential Antidepressant Actions. BioMed Res. Int..

[61] Mechan A.O., Fowler A., Seifert N., Rieger H., Wöhrle T., Etheve S., Wyss A., Schüler G., Colletto B., Kilpert C. (2010). Monoamine reuptake inhibition and mood-enhancing potential of a specified oregano extract. Br. J. Nutr..

[62] Dos Santos É.R.Q., Maia C.S.F., Junior E.A.F., Melo A.S., Pinheiro B., Maia J.G.S. (2018). Linalool-rich essential oils from the Amazon display antidepressant-type effect in rodents. J. Ethnopharmacol..

[63] Zhang L.-L., Yang Z.-Y., Fan G., Ren J.-N., Yin K.J., Pan S.-Y. (2019). Antidepressant-like Effect of Citrus sinensis (L.) Osbeck Essential Oil and Its Main Component Limonene on Mice. J. Agric. Food Chem..

[64] Haleva-Toledo E., Naim M., Zehavi U., Rouseff R. (1999). Formation of α-terpineol in Citrus Juices, Model and Buffer Solutions. J. Food Sci..

[65] Gouveia D., Costa J.S., Oliveira M.A., Rabelo T.K., Silva A., Carvalho A.A., Miguel-Dos-Santos R., Lauton-Santos S., Scotti L., Scotti M.T. (2018). α-Terpineol reduces cancer pain via modulation of oxidative stress and inhibition of iNOS. Biomed. Pharmacother..

[66] Moon P.-D., Choi I.S., Go J.-H., Lee B.-J., Kang S.W., Yoon S., Han S.-J., Nam S.-Y., Oh H.-A., Han N.-R. (2013). Inhibitory Effects of BiRyuChe-Bang on Mast Cell-Mediated Allergic Reactions and Inflammatory Cytokines Production. Am. J. Chin. Med..

[67] Soleimani M., Sheikholeslami M.A., Ghafghazi S., Pouriran R., Parvardeh S. (2019). Analgesic effect of α-terpineol on neuropathic pain induced by chronic constriction injury in rat sciatic nerve: Involvement of spinal microglial cells and inflammatory cytokines. Iran. J. Basic Med. Sci.

[68] Liu S., Zhao Y., Cui H.F., Cao C.Y., Zhang Y.B. (2016). 4-Terpineol exhibits potent in vitro and in vivo anticancer effects in Hep-G2 hepatocellular carcinoma cells by suppressing cell migration and inducing apoptosis and sub-G1 cell cycle arrest. J. Buon.

[69] Agatonovic-Kustrin S., Kustrin E., Morton D.W. (2019). Essential oils and functional herbs for healthy aging. Neural Regen. Res..

[70] Agatonovic-Kustrin S., Chan C.K.Y., Gegechkori V., Morton D.W. (2019). Models for skin and brain penetration of major components from essential oils used in aromatherapy for dementia patients. J. Biomol. Struct. Dyn..

[71] Manayi A., Nabavi S.M., Daglia M., Jafari S. (2016). Natural terpenoids as a promising source for modulation of GABAergic system and treatment of neurological diseases. Pharmacol. Rep..

[72] Chiu G.S., Freund G.G. (2014). Modulation of neuroimmunity by adenosine and its receptors: Metabolism to mental illness. Metab..

[73] Yamada K., Kobayashi M., Kanda T. (2014). Involvement of Adenosine A2A Receptors in Depression and Anxiety. International Review of Neurobiology.

[74] Minor T.R., Hanff T.C. (2015). Adenosine signaling in reserpine-induced depression in rats. Behav. Brain Res..

[75] Peana A.T., Rubattu P., Piga G.G., Fumagalli S., Boatto G., Pippia P., De Montis M.G. (2006). Involvement of adenosine A1 and A2A receptors in (−)-linalool-induced antinociception. Life Sci..

[76] Park H.M., Lee J.H., Yaoyao J., Jun H.J., Lee S.-J. (2011). Limonene, a natural cyclic terpene, is an agonistic ligand for adenosine A2A receptors. Biochem. Biophys. Res. Commun..

[77] Marks D.M., Pae C.-U., Patkar A.A. (2008). Triple Reuptake Inhibitors: The Next Generation of Antidepressants. Curr. Neuropharmacol..

[78] Li B., Zhao J., Lv J., Tang F., Liu L., Sun Z., Wang L., Siwela S.P., Wang Y., Song Y. (2014). Additive antidepressant-like effects of fasting with imipramine via modulation of 5-HT2 receptors in the mice. Prog. Neuro-Psychopharmacology Boil. Psychiatry.

[79] Belujon P., Grace A.A. (2017). Dopamine System Dysregulation in Major Depressive Disorders. Int. J. Neuropsychopharmacol..

[80] Wang J., Jia Y., Li G., Wang B., Zhou T., Zhu L., Chen T., Chen Y. (2018). The Dopamine Receptor D3 Regulates Lipopolysaccharide-Induced Depressive-Like Behavior in Mice. Int. J. Neuropsychopharmacol..

[81] Guzmán-Gutiérrez S.L., Bonilla-Jaime H., Cansino R.G., Reyes-Chilpa R. (2015). Linalool and β-pinene exert their antidepressant-like activity through the monoaminergic pathway. Life Sci..

[82] Karim N., Khan I., Abdelhalim A., Khan A., Halim S.A. (2018). Antidepressant potential of novel flavonoids derivatives from sweet violet (Viola odorata L): Pharmacological, biochemical and computational evidences for possible involvement of serotonergic mechanism. Fitoter..

[83] Abbasi-Maleki S., Mousavi Z. (2017). Hydroethanolic extract of Carthamus tinctorius induces antidepressant-like effects: Modulation by dopaminergic and serotonergic systems in tail suspension test in mice. Iran. J. Basic Med. Sci.

[84] Umukoro S., Adebesin A., Agu G., Omorogbe O., Asehinde S.B. (2017). Antidepressant-like activity of methyl jasmonate involves modulation of monoaminergic pathways in mice. Adv. Med. Sci..

[85] Komiya M., Takeuchi T., Harada E. (2006). Lemon oil vapor causes an anti-stress effect via modulating the 5-HT and DA activities in mice. Behav. Brain Res..

[86] Bassi M.S., Gilio L., Maffei P., Dolcetti E., Bruno A., Buttari F., Centonze D., Iezzi E. (2018). Exploiting the Multifaceted Effects of Cannabinoids on Mood to Boost Their Therapeutic Use Against Anxiety and Depression. Front. Mol. Neurosci..

[87] Gorzalka B.B., Hill M.N. (2011). Putative role of endocannabinoid signaling in the etiology of depression and actions of antidepressants. Prog. Neuro-Psychopharmacology Boil. Psychiatry.

[88] Hill M.N., Hillard C.J., Bambico F.R., Patel S., Gorzalka B.B., Gobbi G. (2009). The Therapeutic Potential of the Endocannabinoid System for the Development of a Novel Class of Antidepressants. Trends Pharmacol. Sci..

[89] Zoppi S., Madrigal J., Caso J.R., García-Gutiérrez M.S., Manzanares J., Leza J.C., Garcia-Bueno B. (2014). Regulatory role of the cannabinoid CB2receptor in stress-induced neuroinflammation in mice. Br. J. Pharmacol..

[90] Zimmermann T., Maroso M., Beer A., Baddenhausen S., Ludewig S., Fan W., Vennin C., Loch S., Berninger B., Hofmann C. (2018). Neural stem cell lineage-specific cannabinoid type-1 receptor regulates neurogenesis and plasticity in the adult mouse hippocampus. Cereb. Cortex.

[91] Abbasi-Maleki S., Kadkhoda Z., Taghizad-Farid R. (2019). The antidepressant-like effects of Origanum majorana essential oil on mice through monoaminergic modulation using the forced swimming test. J. Tradit. Complement. Med..

[92] Coelho V., Mazzardo-Martins L., Martins D.F., Santos A.R., da Silva Brum L.F., Picada J.N., Pereira P. (2013). Neurobehavioral and genotoxic evaluation of (−)-linalool in mice. J. Nat. Med..

[93] Tabari M.A., Moghaddam A.H., Maggi F., Benelli G. (2018). Anxiolytic and antidepressant activities ofPelargonium roseumessential oil on Swiss albino mice: Possible involvement of serotonergic transmission. Phytotherapy Res..

[94] Akbaba E., Hassan S., Sur T.M., Bagci E. (2018). Memory Enhancing, Anxiolytic and Antidepressant Effects of Achillea biebersteinii (Asteraceae) Essential Oil on Scopolamine-Induced Rats. J. Essent. Oil Bear. Plants.

[95] Deyo R., Musty R. (2003). A cannabichromene (CBC) extract alters behavioral despair on the mouse tail suspension test of depression. Proceedings of the 13th Symposium on the cannabinoids, Cornwall, UK, 24 June 2003.

[96] Musty R., Deyo R. (2006). A cannabigerol extract alters behavioral despair in an animal model of depression. Proceedings of the Symposium on the Cannabinoids, Vermont, USA, 24 June 2006.

[97] Cascio M., Gauson L., Stevenson L., Ross R., Pertwee R. (2009). Evidence that the plant cannabinoid cannabigerol is a highly potent α2-adrenoceptor agonist and moderately potent 5HT1A receptor antagonist. Br. J. Pharmacol..

[98] George S.A., Knox D., Curtis A.L., Aldridge J.W., Valentino R.J., Liberzon I. (2012). Altered locus coeruleus-norepinephrine function following single prolonged stress. Eur. J. Neurosci..

[99] Pacak K., Palkovits M., Kvetnansky R., Yadid G., Kopin I., Goldstein D.S. (1995). Effects of Various Stressors on In Vivo Norepinephrine Release in the Hypothalamic Paraventricular Nucleus and on the Pituitary-Adrenocortical Axis. Ann. New York Acad. Sci..

[100] Jaiswal M.K., Seki K., Yoshida S. (2018). Molecular mechanism of noradrenaline during the stress-induced major depressive disorder. Neural Regen. Res..

